# Supplementary material for: Innovation and Utilization of Functional Feed Additives from Maize By-Products in Broiler Chickens
Source: Animals (Basel). 2024 Nov 7;14(22):3198. doi: 10.3390/ani14223198 (PMC11590908; doi:10.3390/ani14223198)
Supplement: Supplementary file 1 [file animals-14-03198-s001.zip › animals-3250381-supplementary.pdf]

Supplementary Table S1. The carcass characteristics in broilers feeding varying levels of spent mushroom substrate.

Item	CON	AGP [1]	SMS0.5	SMS1.0	SMS2.0	SEM	*p*-value		
							T	L	Q
Live weight (g)	1934.00 ^{b}	2232.00 ^{a}	2088.00 ^{a}	2196.00 ^{a}	2102.00 ^{a}	28.893	0.003	<0.05	0.365
Defeather weight (g)	1822.00 ^{c}	2123.80 ^{a}	1960.00 ^{b}	2065.60 ^{a,b}	2016.40 ^{a,b}	28.102	0.002	<0.05.	0.314
Carcass weight (g)	1585.20 ^{c}	1863.60 ^{a}	1722.40 ^{b}	1821.20 ^{a,b}	1761.20 ^{a,b}	25.178	0.001	<0.05	0.267
Carcass percentage (%)	81.95 ^{c}	83.50 ^{a,b}	82.51 ^{b,c}	82.96 ^{a,b,c}	83.79 ^{a}	0.197	0.009	0.471	0.255
Carcass composition (% lw)									
Neck	3.32	2.89	2.94	3.26	3.37	0.082	0.200	0.626	0.266
Head	2.49	2.19	2.18	2.29	2.63	0.062	0.068	0.156	0.606
Wing	8.19	7.33	7.64	7.46	7.79	0.105	0.067	<0.05	0.390
Drumstick	10.47	10.06	9.91	10.00	10.51	0.098	0.150	0.070	0.471
Shank	3.77	3.57	3.62	3.68	3.80	0.041	0.366	0.347	0.574
Skeleton	19.99	20.98	22.01	21.21	21.46	0.228	0.058	0.113	<0.05
Meat percentage (% lw)									
Breast	23.04	24.83	23.91	24.44	23.95	0.218	0.091	0.067	0.543
Thigh	8.71	8.25	8.17	8.24	8.01	0.119	0.445	0.436	0.177
Inner organ percentage (% lw)									
Liver	2.13	1.98	2.24	2.29	2.07	0.040	0.074	0.072	0.872
Spleen	0.16	0.13	0.15	0.15	0.18	0.010	0.643	0.512	0.622
Kidney	0.45	0.56	0.63	0.40	0.53	0.034	0.227	0.621	0.056
Heart	0.54	0.57	0.48	0.52	0.53	0.012	0.267	0.513	0.229
Lung	0.54	0.60	0.58	0.41	0.65	0.031	0.152	0.117	0.127
Proventriculus	0.63	0.58	0.48	0.44	0.59	0.043	0.619	0.073	0.663
Gizzard	2.44	2.03	2.30	1.88	2.27	0.076	0.121	<0.05	0.634
Small intestine	2.31	2.18	2.53	2.46	2.49	0.053	0.186	0.477	0.283
Ceacum	0.81	0.81	0.87	0.74	0.87	0.021	0.220	0.264	0.062
Abdominal fat	0.65	0.96	0.71	0.75	0.94	0.053	0.221	0.986	0.447

[1] Antibiotic (amoxicillin and colistin at 0.25 g kg^{-1}); SMS0.5, spent mushroom substrate addition at 0.5 g kg^{-1}; SMS1.0, spent mushroom substrate addition at 1.0 g kg^{-1}; SMS2.0, spent mushroom substrate addition at 2.0 g kg^{-1}. [a,b,c] Mean values in the same row with different letters indicate significant differences (*p*-value < 0.05). SEM, standard error of the mean. T is the *p*-value of the effect of treatment on analyzed traits; L and Q are the *p*-value of the linear and quadratic contrast of SMS supplementation.

Supplementary Table S2. The meat quality in broilers feeding varying levels of spent mushroom substrate.

Item	CON	AGP [1]	SMS0.5	SMS1.0	SMS2.0	SEM	*p*-value		
							T	L	Q
pH 0 h	6.97	6.82	6.86	6.92	6.92	0.053	0.921	0.909	0.678
pH 24 h	6.73	6.84	6.82	6.92	6.72	0.055	0.811	0.939	0.238
Breast									
Lightness; L*	54.89	54.82	54.55	54.59	53.56	0.362	0.811	0.277	0.790
Redness; a*	6.14	5.85	6.15	6.25	5.80	0.417	0.997	0.020	<0.001
Yellowness; b*	13.86	13.88	14.23	14.31	13.26	0.241	0.705	<0.05	<0.001
Drip loss (%)	1.40	1.61	1.53	1.45	1.47	0.087	0.966	<0.05	<0.001
Shear force (kgf/cm^2)	3.30	3.29	3.30	3.33	3.30	0.007	0.313	0.704	0.149
Cooking loss (%)	25.54	25.51	25.56	25.59	25.45	0.025	0.461	0.182	0.180
Thigh									
Lightness; L*	49.62	50.47	50.20	50.55	49.08	0.345	0.663	0.086	<0.05
Redness; a*	7.72	8.55	7.84	8.88	7.57	0.600	0.958	<0.05	<0.05
Yellowness; b*	12.13	11.91	11.47	12.37	11.37	0.288	0.810	0.347	0.584
Shear force (kgf/cm^2)	4.10	4.10	4.10	4.13	4.11	0.006	0.283	0.262	0.279
Cooking loss (%)	20.14	20.08	20.12	20.17	20.03	0.024	0.454	0.166	0.282

[1] Antibiotic (amoxicillin and colistin at 0.25 g kg^{-1}); SMS0.5, spent mushroom substrate addition at 0.5 g kg^{-1}; SMS1.0, spent mushroom substrate addition at 1.0 g kg^{-1}; SMS2.0, spent mushroom substrate addition at 2.0 g kg^{-1}. Mean values in the same row with different letters indicate do not significant differences (*p*-value > 0.05). SEM, standard error of the mean. T is the *p*-value of the effect of treatment on analyzed traits; L and Q are the *p*-value of the linear and quadratic contrast of SMS supplementation.
